# Reactive Astrocytes in Neurodegenerative Diseases

**DOI:** 10.14336/AD.2018.0720

**Published:** 2019-06-01

**Authors:** Kunyu Li, Jiatong Li, Jialin Zheng, Song Qin

**Affiliations:** ^1^Department of Anatomy, Histology and Embryology, School of Basic Medical Sciences, Fudan University, Shanghai 200032, China.; ^2^Center for Translational Neurodegeneration and Regenerative Therapy, Shanghai Tenth People’s Hospital affiliated to Tongji University School of Medicine, Shanghai, China.

**Keywords:** reactive astrocytes, neuroinflammation, neurodegenerative diseases

## Abstract

Astrocytes, the largest and most numerous glial cells in the central nervous system (CNS), play a variety of important roles in regulating homeostasis, increasing synaptic plasticity and providing neuroprotection, thus helping to maintain normal brain function. At the same time, astrocytes can participate in the inflammatory response and play a key role in the progression of neurodegenerative diseases. Reactive astrocytes are strongly induced by numerous pathological conditions in the CNS. Astrocyte reactivity is initially characterized by hypertrophy of soma and processes, triggered by different molecules. Recent studies have demonstrated that neuroinflammation and ischemia can elicit two different types of reactive astrocytes, termed A1s and A2s. However, in the case of astrocyte reactivity in different neurodegenerative diseases, the recently published research issues remain a high level of conflict and controversy. So far, we still know very little about whether and how the function or reactivity of astrocytes changes in the progression of different neurodegenerative diseases. In this review, we aimed to briefly discuss recent studies highlighting the complex contribution of astrocytes in the process of various neurodegenerative diseases, which may provide us with new prospects for the development of an excellent therapeutic target for neurodegenerative diseases.

Astrocytes are the most abundant cells with various structures and functions and are ubiquitous in all regions of the central nervous system (CNS). As is well characterized and reviewed in many monographs, astrocytes are associated with various aspects of physiological functions, including secretion of nutrients, maintenance of neuronal microenvironment, regulation of the permeability of the blood-brain barrier and the development of pathological processes in the brain [1]. In the past few decades, researchers have provided a great deal of understanding towards the role of astrocytes in the CNS physiology and disease, in which the interactive signaling networks between neurons and other cell types have attracted much attention [2].

It is widely believed that astrocytes can guide the migration of postnatal neuroblasts and promote functional coordination of the brain by intimate contact with the entire region of the brain [3, 4]. More importantly, they often play a key role in regulating homeostasis, as well as the secretion and metabolism of amino-acid-based neurotransmitters such as glutamate acid and gamma-amino butyric acid (GABA) [5, 6]. Astrocytes can receive a variety of substances and signals through the conduction of various receptors and signaling pathways. In this way, they can affect the uptake and synthesis of neurotransmitters or neurotrophic factors through synaptic connections with neurons. In addition, astrocytes are involved in regulating the innate immune response by regulating inflammatory factors, such as cytokines, chemokines, complement proteins and reactive oxygen species or reactive nitrogen species [7].

A large amount of studies on mouse models have shown that astrocytes play a complex role in the pathogenesis of neurodegenerative diseases, and the dysfunction of astrocytes may contribute to either neuronal death or the process of neural disturbances [8, 9]. It has been found that reactive astrocytes always lose their supportive role and gain toxic function in the progression of neurodegenerative diseases[9, 10]. Therefore, in this article we will briefly describe how reactive astrocytes respond to injuries in the CNS during the process of neurological disorders caused by neurodegenerative diseases, and the role of reactive astrocytes in different neurodegenerative diseases, such as Alzheimer’s disease (AD), Parkinson’s disease (PD), Amyotrophic Lateral Sclerosis (ALS) and Multiple Sclerosis (MS).

## Astrocytes morphology and functions in the CNS

Astrocytes are the most widely distributed cells in the CNS of mammals [11], which emit many long branching extensions from the cell body, filling the space between the cell body and their expansion. Endfeet are usually formed at the extended ends of the astrocytes, where those connected to the capillary wall are called the perivascular feet. Unlike neurons, astrocytes do not have Nissl substances in the cytoplasm; instead, they contain a large number of interlaced fibrils, extending in parallel to the cellular processes and constituting the main component of the astrocytes skeleton. The ultrastructure of these fibers is called glial filament, which is a kind of intermediate filaments with a diameter between microtubules and microfilaments [2]. Glial filaments are composed of glial fibrillary acid protein (GFAP), which, together with Vimentin acts as a main factor for the intermediate filaments constituting the cytoskeleton of astrocytes [11, 12].

**Figure 1.**
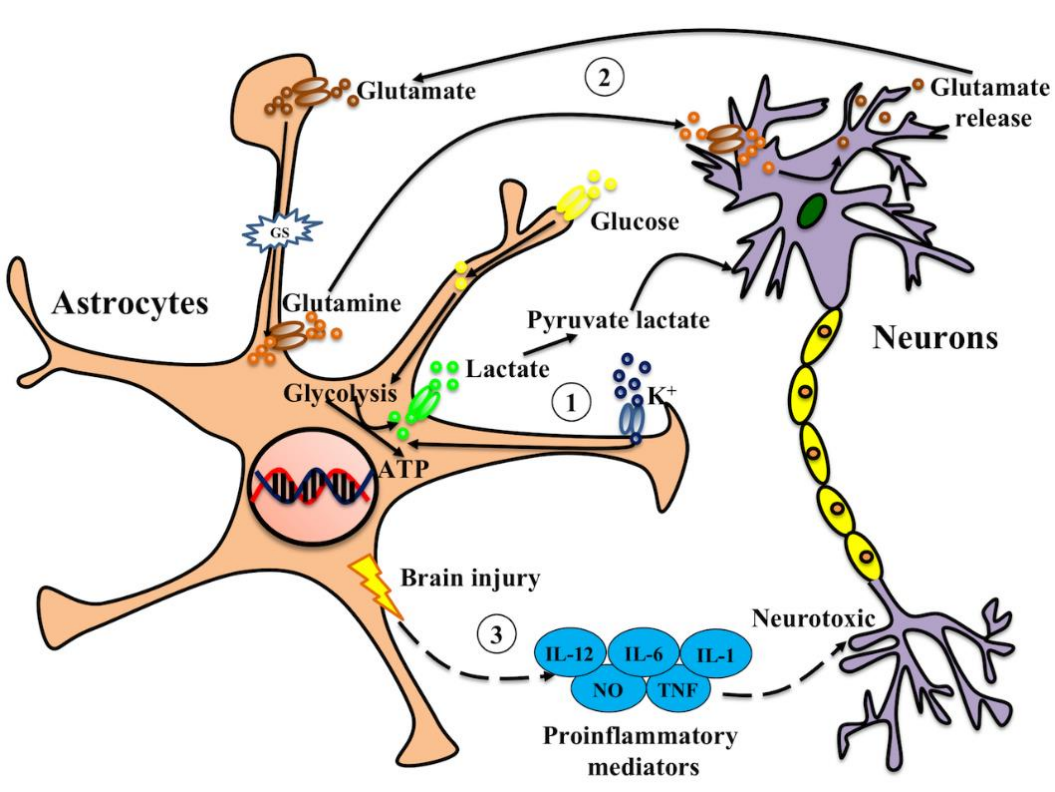
Astrocytes play a critical role in supporting neurons in the CNS Astrocytes support neuronal functions in multiple ways. Indeed, the extracellular levels of ions and neurotransmitters can affect the excitability of neurons. (1) High concentrations of extracellular potassium can trigger the glycolysis of astrocytes, which can enhance the release of lactate and pyruvate, thereby supporting neuronal metabolism. (2) Astrocytes can take up glutamate and convert it to glutamine, which is then released into the extracellular space and taken up by neurons to resynthesize glutamate. Any deregulation of these mechanisms is a common condition for neurodegenerative diseases. (3) Under the circumstances of brain injury, disease or inflammatory insult, toxic proinflammatory mediators are secreted and released by astrocytes, which will act on neurons and may affect the survival of neurons.

**Table 1:** The molecular expression changes between two different types of reactive astrocytes.

Reactive astrocytes	Molecular expression		Refs
A1 astrocytes	**Up** **regulation**	Inflammatory signaling through NF-κB	^[105, 106]^
		Glutamate and ATP release	^[107, 108]^
		Inflammatory mediators secretion (prostaglandinD2, IFN-γ, and TGF-β)	^[93, 109]^
		Lcn2 secretion	^[110]^
		IL-1α, C1q, TNF	^[9, 10]^
	**Down** **regulation**	GPC4, GPC6, SPARCL1 expression	^[111, 112]^
		Excitatory amino acid transporter 2 (EAAT2), Glutamate transporter 1 (GLT1)	^[113, 114]^
		Trophic factor release	^[9, 115]^
		Lactate transportation	^[85]^
		GABA release through GAT-3	^[116]^
A2 astrocytes	**Up** **regulation**	Inflammatory signaling through STAT3	^[27, 29]^
		Thrombospondins (THBS1 and THBS2)	^[33]^
		Aquaporin-4	^[34]^
		HMGB1 and β-2 integrin	^[35]^
		Trophic factor release (BDNF, VEGF and bFGF)	^[9, 36, 37]^
	**Down** **regulation**	H2-D1, Gbp2, Fkbp5, Srgn	^[10]^

Astrocytes can be divided into two major sub-types, fibrous or protoplasmic astrocytes, based on the differences in their cellular morphology as well as the content of glial filaments. Located mainly in the white matter, fibrous astrocytes exhibit morphology with numerous long fiber-like processes and contain many glial filaments in the cytoplasm. Protoplasmic astrocytes are widely distributed in the grey matter, showing highly bushy branches [2, 13]. In addition, some special types of astrocytes have been discovered in adult brain, such as radial glial cells, Müller cells, and Bergmann glial cells, which can be distinguished morphologically in different brain regions [2, 14].

Astrocytes also mediate the formation of neurovascular unit through their processes, acting as a bridge between neurons and blood vessels. The astrocytic endfeet closely surround endothelial cells and pericytes, which is crucial for the structure of blood-brain barrier [15, 16]. By increasing tight junctions and reducing gap junctions, they can affect the integrity of blood-brain barrier. In addition, astrocytes can provide structural and metabolic support for neurons [17, 18], and play a vital role in regulating neuronal survival, morphology[3], axon growth [19], synapse formation [20], and the distribution of ion channels (Fig. 1). Through their receptors, astrocytes can assist in the activation of calcium ions by sensing changes in neurotransmitter or extracellular environment, leading to the release of neurotransmitters (such as glutamine), which in turn affects synaptic transmission [6, 11, 21]. Astrocytes are the main antigen-presenting cells in the brain, and their cell membranes exhibit major histocompatibility complexes. The major histocompatibility complex (MHC) is known to function in antigen processing by combining and processing foreign antigens and providing them to T lymphocytes, which indicates that astrocytes may have some association with autoimmune diseases [22]. Recently researchers have found that in the process of ischemia and traumatic injuries, the pathophysiological changes in the CNS and neurodegeneration symptoms are mainly attributed to the loss of normal functions of astrocytes [23-25]. During brain insult or neurodegeneration process, astrocytes can respond to pathological changes by releasing extracellular molecules, such as neurotrophic factors (for example BDNF, VEGF and bFGF), inflammatory factors (including IL-1β, TNF-α and NO, etc.) and cytotoxins (such as Lcn2) through reactive astrogliosis. As a result, they play either a neuroprotective or neurotoxic role (such as provoking inflammation or increasing damages) in the CNS [23, 26].

**Figure 2.**
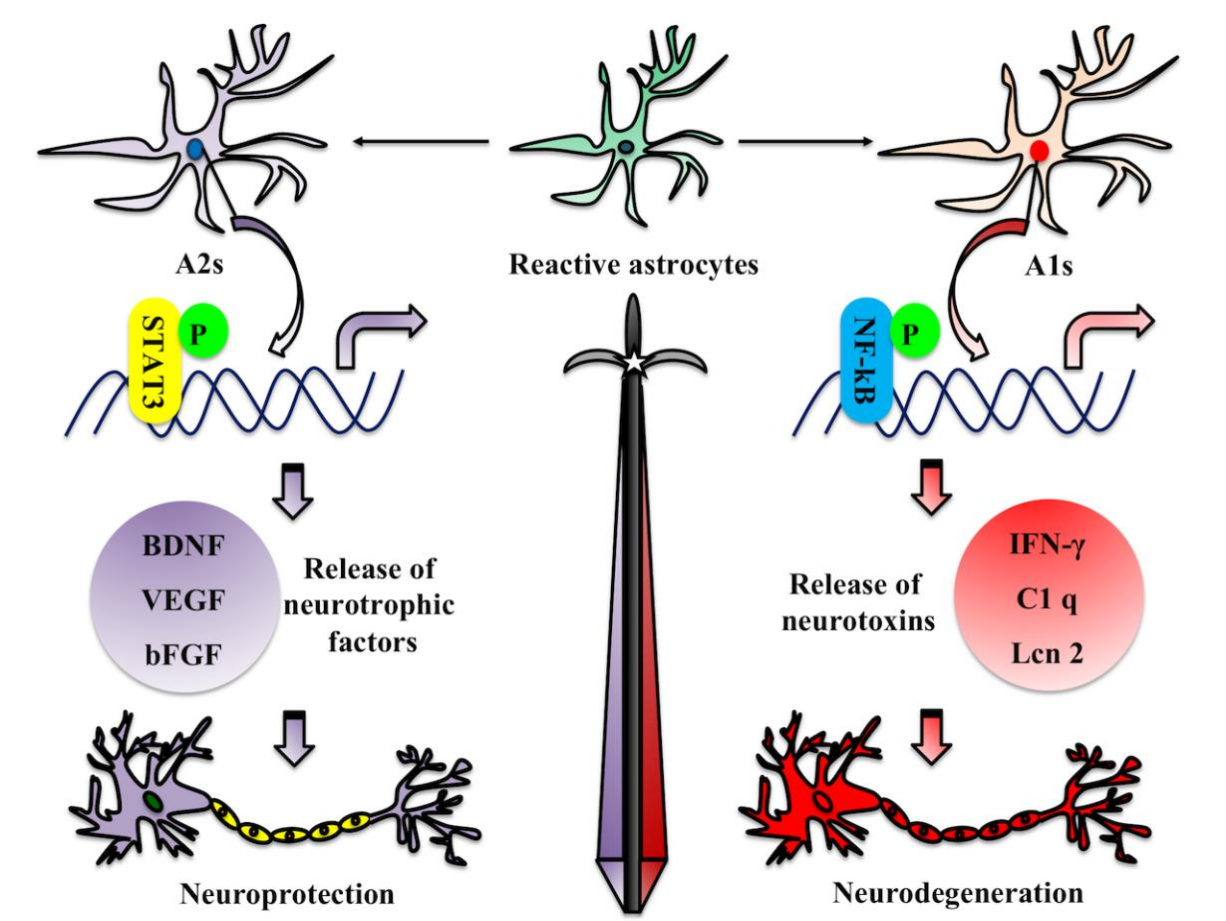
Roles of reactive astrocytes in the process of neuroinflammation or brain injury Neuroinflammation mainly induces the formation of A1 reactive astrocytes (A1s), which exhibit differential expression of astrocytic receptors, transporters, transmitters, as well as the changes of protein release and inflammatory factors. These changes may result in loss of neuroprotective function or neurological toxicity, a collapse of the brain-blood barrier and an increase in inflammation of the brain, which eventually results in deaths of neurons and causes neurodegenerative diseases. While A1s can upregulate many genes that are destructive to synapses, A2 reactive astrocytes (A2s) can upregulate many neurotrophic factors promoting the survival of neurons.

It has been shown that the specific deletion of STAT3 in astrocytes can cause reactive gliosis, which leads to increased level of inflammation, tissue damage as well as compromised motor recovery after spinal cord injury [27-29]. Interestingly, some studies have shown that the activation of NF-κB in astrocytes contributes to the pathogenesis of CNS, and inhibition of this signaling pathway can limit tissue damage [30-32]. These findings suggest that astrocytes may play a protective role through STAT3 signaling pathways in some neurodegenerative lesions, while NF-κB signals may mediate neurotoxicity. In analogy to the “M1” and “M2” phenotype categories for macrophages, recent studies have reported that neural inflammation and ischemia can induce two types of reactive astrocytes, termed “A1” and “A2”, respectively [9, 10]. Gene transcriptome analysis of reactive astrocytes shows that A1 reactive astrocytes (A1s) can upregulate many classical complement cascade genes that are destructive to synapses, and secret neurotoxins that have not yet been well identified[9]. In contrast, A2 reactive astrocytes (A2s) can upregulate many neurotrophic factors [33-37], which can promote either the survival and growth of neurons or the synaptic repair (Table 1). Thus, A1s may have “harmful” features, while A2s may carry “useful” or repair functions (Fig. 2). So far, it remains unclear what the possible signaling pathways have been involved in inducing the phenotypes of A1s and A2s in the process of different initiating CNS injuries.

Recent studies have also shown that in occurrence and development of neurodegenerative diseases, the expression level of GFAP, Vimentin and calcium-binding protein S100β in astrocytes is increased [38]. Remarkably, for most patients with neurodegenerative disease, reactive astrocytes are ubiquitous in the CNS tissues [39, 40]. Further study on the role of reactive astrocytes in different types of neurodegenerative diseases, and their alteration of effects in different neurodegenerative states may shed light on future prevention and treatment of these diseases.

### Astrocytic responses in neurodegenerative diseases

Neurodegenerative disease is a disorder that could affect and cause pathological changes throughout the mammalian nervous system, especially in the cerebral cortex and the basal ganglia [41]. The causes of these diseases are complex and still remain unknown, due to both genetic and sporadic issues. Neuropathological and radiological studies have shown that these diseases are accompanied by serious inflammation, during which the glial response may be a significant issue in the occurrence of neurons losses [42, 43]. Studies based on molecular biology show that the causes of neurodegenerative disorders are multi-systemic in vivo, and may be affected by a cascade of multiple signaling pathways [44]. In this section, we will briefly discuss the above issues and focus on the role of reactive astrocytes in the pathological process of neurodegenerative diseases.

#### Alzheimer’s disease (AD)

Alzheimer’s disease (AD) is one of the most common neurodegenerative diseases of the CNS, characterized by memory loss and cognitive dysfunction, and has a variety of neurological and psychiatric symptoms and behavioral disorders [45]. Pathologically, AD is marked by the presence of extracellular amyloid plaques (APs) and intracellular neurofibrillary tangles (NFTs) in the brain. The APs are composed of aggregated β-amyloid peptide (Aβ), whereas the NTFs are composed of abnormal phosphorylation and aggregation of tau protein intracellularly [7, 46]. The cause of AD is thought to be impairment of the relationship between neuronal and astrocytic functions in brain regions associated with memory or thinking (such as hippocampus) [7].

Under normal circumstances, the precursor protein of amyloid (APP) is mainly cleaved by secretase α, producing sAPPα - a soluble neuroprotective fragment and blocking the production of Aβ, thereby protecting brain cells from the toxic damage of Aβ[47, 48]. In AD condition, however, the cleavage is mainly completed by secretase β and secretase γ. It subsequently forms Aβ primarily, which can be divided into Aβ40 and Aβ42 according to the length of the peptide segment [47].

With the development of AD, the reduction of Aβ clearance can aggravate Aβ plague formation, possibly due to the dysfunction of astrocytes and the formation of Aβ [40, 49]. The study on AD mouse model found that the Aβ plaques could be removed by transplanting the fluorescent-labeled wild-type astrocytes [50], indicating that astrocytes could play a role in the removal of Aβ peptide in normal brain. Another study also confirmed strong β-site APP cleaving enzyme 1 (BACE1) expression in reactive astrocytes of AD patients, which can contribute to the production of Aβ [51]. Further investigation is needed to examine the process and amount of Aβ produced in astrocytes compares with neurons.

The abnormal regulation of calcium and glutamate homeostasis in reactive astrocytes may lead to the pathogenesis of AD [52]. In mouse model of AD brain, the astrocytic calcium signaling and gliotransmitter releases can be disrupted by Aβ, which indicates astrocyte dysfunction may contribute to the earliest neuronal deficits in AD [53]. In hippocampus of AD mice, excessive GABA released by reactive astrocytes can result in tonic inhibition of dentate gyrus granule cells in hippocampus of AD mice. Remarkably, inhibition of GABA synthesis or pharmacological blockade of GABA transporters restores synaptic plasticity and memory deficits in these mice [54].

Upregulation of intermediate filament is a hallmark of astrocytes reactivity in AD brain [55]. Pathological studies using AD brain samples and mouse models have found that the APs are surrounded by reactive astrocytes, with an increased expression of GFAP and S100β [56, 57]. Moreover, the degree of the number increased of reactive astrocytes is often correlated with cognitive decline[58]. In normal situations, reactive astrocytes are the main regulators in brain’s inflammatory response, but under pathological conditions in the brain, reactive astrocytes may be neurotoxic when producing inflammatory cytokines and reactive oxygen species [59]. In AD brains, the levels of pro-inflammatory cytokines produced mainly by reactive astrocytes are high in regions where reactive astrocytes accumulate [60]. Increased levels of pro-inflammatory cytokines and activated inflammasomes production were also observed in brain tissue of AD patients [61]. Transcriptional analysis also revealed higher levels of these inflammatory factors in astrocytes than in microglia [62]. In addition, aberrant energy metabolism was also observed in AD reactive astrocytes. By blocking the energy metabolism and oxidative stress in AD astrocytes, the effect of Aβ plaque deposition can be reduced, thus improving memory and delaying disease progression [63].

The expression of APP has been shown in astrocytes, and its expression level is upregulated by multiple pro-inflammatory cytokines (IL-1β and IL-6) in mouse brain[64]. The pro-inflammatory cytokine combinations markedly increase the expression of APP and secretase β in the primary cultured astrocytes [65]. Interestingly, traumatic brain injury has long been associated with the risk of developing AD [66]. Several lines of evidence have shown that acute brain injury can induce APP and PS1 expression in reactive astrocytes [67, 68]. These studies indicate the reactive astrocytes contribute to Aβ production in AD. However, whether there exists a neuroprotective role of reactive astrocytes in different stages of AD needs further investigation.

#### Parkinson’s disease (PD)

Parkinson’s disease is a progressive neurodegenerative disorder caused by the disruption of dopaminergic neurotransmission in the basal ganglia and neuronal death in the substantia nigra (SN). The pathological hallmark of PD is the presence of α-synuclein deposition and protein inclusions also known as Lewy bodies or Lewy neurites in neuronal cell cytoplasm [69, 70]. So far, there still lacks molecular mechanisms to fully understand the pathogenesis of PD, and there is no clinically effective treatment for the disease. Previous studies have shown that mutations in various proteins, such as PARK2, ATP13A2, PTEN, PINK1 and DJ-1, can lead to PD-like symptoms [71-73]. Studies in vivo found that intravenous injection of 1-Methy-4phenyl-1, 2, 3, 6-tetrahydro-pyridine (MPTP) can lead to Parkinsonism in mice. This is due to the presence of a monoamine oxidase enzyme in the cytoplasm of astrocytes, which can transfer MPTP to MPP^+^, while MPP^+^ can kill or damage dopaminergic neurons, leading to paralysis agitans [74, 75].

Astrocyte reactivity is detected in the SN pars compacta (SNpc) of patients with PD[76]. At the time of PD initiation, α-synuclein accumulated in astrocytes, which subsequently led to recruitment of phagocytic microglia, attacking certain neurons in the restricted brain region and causing the clinical symptoms of PD [77]. Pathological examinations of PD brains show an increased number of astrocytes as well as an elevated level of GFAP expression [78]. Pathological studies also found that astrocytes can be activated and accumulated with nonfibrillized α-synuclein at early stages of PD brain, which distributed more broadly than Lewy bodies [79, 80]. Interestingly, the increasing accumulation of α-synuclein aggregates was found in pre-symptomatic and symptomatic mouse brains and correlated with the expansion of reactive astrogliosis[81]. The presence of intracellular aggregates may disrupt astrocytic glutamate transporters and their ability to regulate blood-brain barrier, leading to non-cell-autonomously damaging neurons [81]. The above findings suggest that the reciprocal communication between astrocytes and neurons is of great significance to the health of PD neurons.

Similar to the characteristics of reactive astrocytes in AD mouse models, the activation of STAT3 signaling pathway in astrocytes seems to be a consistent feature in PD. Pharmacological inhibition of JAK2 in MPTP mouse model of PD can significantly result in the decrease of pSTAT3 and GFAP expression levels and reactivity of astrocytes, suggesting that the JAK/STAT3 signaling pathway is required to induce reactivity of astrocytes in the diseased condition [82].

In normal circumstances, Fzd-1 receptor in mesencephalic neurons is required for astrocyte-mediated neuroprotection [83]. Studies in mesencephalic neurons have discovered that the Fzd/β-catenin signaling pathway mediated by antagonist can inhibit neuronal survival in SN-induced reactive astrocytes, which can be prevented by pharmacological activation of β-catenin within the SN [83]. These results show that the Wnt1/Fzd-1/β-catenin signaling pathway plays a key role in the interaction between astrocytes and neurons and is of vital importance for maintaining the health of PD neurons.

#### Amyotrophic Lateral Syndrome (ALS)

Amyotrophic Lateral Syndrome (ALS) is an irreversible progressive motor neuron disease characterized by degeneration of motor neurons in the CNS, which results in muscle atrophy or even death caused by respiratory failure [84]. The fundamental pathological basis for ALS remains to be investigated. In sporadic ALS cases, there is no clear genetic component involved in the process of the disease. However, in the case of inherited ALS, gene mutations result in aggregated forms of proteins (such as SOD1), which are found in both neurons and astrocytes [85].

Reactive astrocytes are observed in vulnerable regions and the degree of reactivity correlates with the neurodegeneration level in ALS patients. Moreover, the only insult discovered until now is the death of specific category of motor neurons. ALS astrocytes have been shown to directly contribute to motor neuron death in vivo. At the same time, these astrocytes are accompanied with various abnormalities of signaling pathways, such as changes in neuronal expression of GluR2 subunit of AMPA receptors [86], impaired lactate transport [85], activation of p75-receptor signaling in motor neurons [85], reduction of GLT-1 expression and persistent Ca^2+^ release and apoptosis resulted from mGluR5-mediated glutamate signaling [87].

One study of mouse models revealed that transplantation of the precursors of mutant SOD1 astrocytes into the spinal cords can lead to degeneration of motor neurons [88], whereas transplantation of wild type astrocytic precursors into ALS mouse models results in the decrease of motor neurons death, which indicates that ALS astrocytes have harmful or toxic effects on motor neurons in vivo [89]. Consistently, another in vivo study using the SOD1 (G93A) mouse model found that astrocytes may have defective glutamate uptake, resulting in extracellular accumulation of glutamate, which is toxic to motor neurons [90]. In contrast, by increasing the activation of astrocytes and the expression of immune or inflammatory markers in vivo, the illusory pathological process can be exacerbated [91].

In ALS, a major increase in the transcription of inflammatory molecules is well established, including those in astrocytes derived from both familial and sporadic forms of the disease [92]. The pathological process of ALS is hallmarked by the activation of astrocytes and the expression of immune or inflammatory markers in vivo. In addition, several inflammatory mediators (in particular IL-6 and TGFβ) are found to participate in astrocyte-neuron communication [5, 93]. For example, it has been shown that interferon-γ induced reactive astrocytes may be neurotoxic [94], possibly via STAT3-dependent signaling pathway, in which activation of STAT3 results in the recruitment of reactive astrocytes and the response of reactive microglia to motor neurons [28]. Importantly, both the recruitment and the neurotoxicity of reactive astrocytes in ALS can be inhibited by STAT3 inhibitors [95].

**Figure 3.**
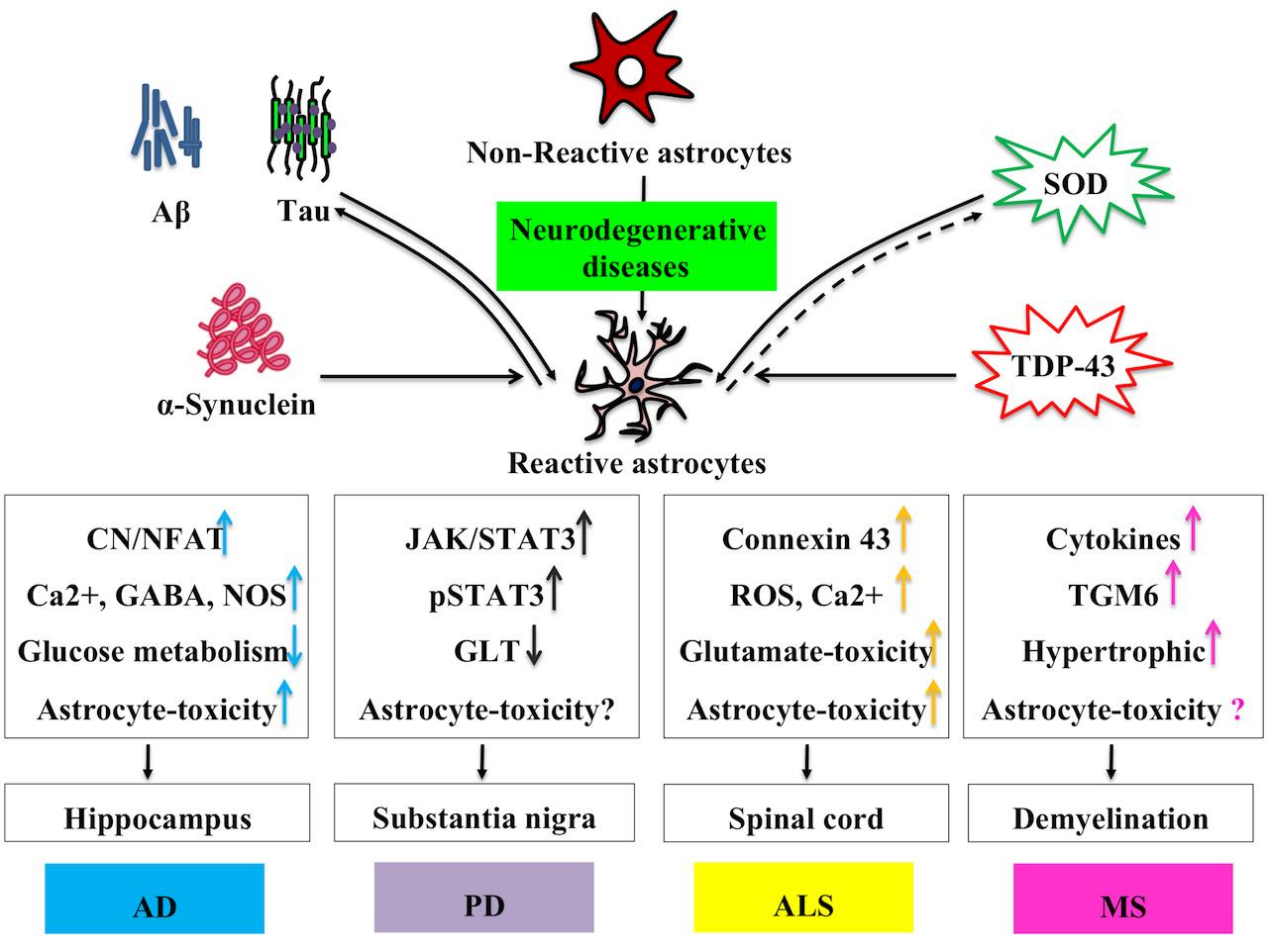
Characteristics of reactive astrocytes in different neurodegenerative diseases Various molecules can trigger the reactivity of astrocytes, which involves their morphological, transcriptional and functional changes. Different neurodegenerative diseases lead to a variety of changes in reactive astrocytes, which may ultimately cause them to release fewer neurotrophic factors and produce more inflammatory factors. This effect largely depends on different neurodegeneration-related factors, and the molecules they produce and secrete into the microenvironment surrounding the functional neurons in the brain. Aβ, amyloid β; SOD, superoxide dismutase-1; TDP-43, TAR DNA-binding protein 43; CN/NFAT, Calcineurin/Nuclear factor of activated T-cells; NOS, Nitric Oxide Synthase; JAK, Janus Kinase; ROS, reactive oxygen species; TGM6, Transglutaminase 6

#### Multiple Sclerosis (MS)

Multiple Sclerosis (MS) is an inflammatory disorder that leads to demyelination and axonal injury in CNS. Although its etiology remains elusive, there is some evidence supporting the concept that autoimmunity plays a major role in the pathogenesis of this disease [96]. The activity of astrocytes was widely spread around acute inflammatory lesions in the MS brain [97]. Murine experimental autoimmune encephalomyelitis (EAE) is an established animal model of multiple sclerosis, which shows the activation of astrocytes, accompanied by the loss of endfeet around small blood vessels, the loss of BBB function, subsequently followed by CNS inflammation and perivascular edema [97]. Consistently, pathological examination revealed the presence of reactive astrocytes in patient with acute MS [97]. It is reported that genomic changes can be found in astrocytes from stroke and inflammatory lesions [98]. The researchers also found that reactive astrocytes lack the expression of MHC-Ⅱtrans-activators, leading to the failure to activate CD4^+^ T cells, which suggests that MHC expression plays a key role in the activation of T cells in astrocytes [99]. In addition, reactive astrocytes combined with VEGFR-2 can activate the expression of vascular endothelial growth factor and PLC-γ1, downregulate the expression of claudin-5 and occluding, and eventually break the BBB [100]. In MS brain, reactive astrocytes are also the main source of inflammatory cytokines (including IL-10, IL-17A, IL-22 and MIP-1α, etc.), which may trigger uncontrolled inflammatory reactions that lead to the activation of T cells and the formation of myelin sheath in focal areas and axonal damage [101-103]. However, whether there are distinctive reactive astrocytes contributing to Multiple Sclerosis is still unclear, therefore further investigation is needed.

### Conclusions

Reactive astrocytes play a complex role under different pathological situations. In the process of neuro-degenerative diseases, astrocytes become responsive and dramatically alter their phenotypes. Recently, one study by Chen et al. has found that astrocyte-specific deletion of Sox2 in adult mice greatly diminishes glial response to controlled cortical impact injury, and benefits behavioral recovery of mice after traumatic brain injury [104]. These data strongly suggest that Sox2-dependent pathways in reactive astrocytes may be specifically targeted for brain recovery after injury.

Two distinct phenotypes of reactive astrocyte have been identified in mice, depending on the initiation injury, in which A1s are mainly induced by LPS-induced inflammation, whereas A2s are mainly caused by ischemia. As described in this review, in the neurodegenerative states, such as AD, PD, ALS and MS, reactive astrocytes can involve both neuroprotective and neurodegenerative functions. This effect depends largely on the molecules they produce and secrete into the microenvironment around the neurons (Fig. 3). So far, however, the understanding of the role of reactive astrocytes is still in its infancy. With all the studies mentioned above, how to regulate the function of reactive astrocytes and develop them to obtain “useful” features may be an exciting point. Progress in this field will not only bring prosperity to the further understanding and treatment of neurodegenerative diseases but will undoubtedly pave the way for effective clinical strategies for intractable neurological diseases as well.
